# Does X‐ray imaging by GPC at emergency care access points in the Netherlands change patient flow and reduce ED crowding? A cohort study

**DOI:** 10.1002/hsr2.26

**Published:** 2018-02-08

**Authors:** D.L.C.M. van den Bersselaar, M. Maas, W.A.M.H. Thijssen

**Affiliations:** ^1^ Catharina Hospital Eindhoven the Netherlands

**Keywords:** after‐hours care, crowding, emergency department, general practitioner cooperative, primary health care, X‐rays

## Abstract

**Objective:**

Organizing out‐of‐hours emergency care is a challenge in many countries. In the Netherlands, general practitioner cooperatives (GPCs) and emergency departments (EDs) are increasingly working together, creating one emergency care access point (ECAP). This has redirected the majority of patients with musculoskeletal problems from the ED to the GPC in out‐of‐hours care, due to the treatment of self‐referrals by the general practitioner (GP). Only a minority of the GPs at ECAPs have the possibility to request X‐rays, and expanding these facilities could reduce patient presentations to the ED even more. The aim of our study was to explore patient flow and possible reductions in ED referrals at an ECAP with X‐ray facilities for GPs.

**Methods:**

This retrospective cohort study examines all patients that visited an ECAP at a general city hospital in the Netherlands and had an X‐ray imaging requested by the GPC between January 1, 2014 and December 31, 2014. General practitioner cooperatives could request X‐rays between 5 pm and 10 pm on weekdays and between 8 am and 10 pm during weekends. Recorded data included sex, age, number and type of X‐ray, X‐ray abnormalities, referral to the ED, and treatment. The annual number of patients presenting to the GPC and ED in 2014 were gathered. Patient outcome was stated negative when the X‐ray revealed no abnormality.

**Results:**

A total of 2243 patients received 2663 X‐ray examinations. The mean age was 31 years and 48% was male. A total of 1517 (68%) patients were treated at the GPC without an ED referral, a reduction of 4.5% of the annual ED patients.

**Conclusions:**

With a majority (68%) of the patients examined and treated at the GPC, X‐ray facilities at ECAPs will substantially reduce ED population, change patient flow, and have a positive effect on ED crowding. Implementing 24/7 X‐ray facilities at all ECAPs will further enhance these effects.

## Abstract 2

Organizing out‐of‐hours emergency care is an important challenge in many countries. EDs are confronted with crowding, while access to X‐ray facilities for general practitioners in out‐of‐hours care is minimal, leading to unnecessary referrals. This study showed that X‐ray facilities for general practitioner cooperatives in out‐of‐hours care will substantially reduce ED population, change patient flow, and have a positive effect on ED crowding.

**Abbreviations**
ECAP: Emergency Care Access Point
ED: Emergency Department
GP: General Practitioner
GPC: General Practitioner Cooperative
NTS: Netherlands Triage System

## 1 INTRODUCTION

Organizing out‐of‐hours emergency care is an important challenge in many countries. Since 2000, primary care settings in many European countries have changed from rota‐groups towards general practitioner cooperatives (GPCs).^1^, ^2^ In the Netherlands, GPCs and emergency departments (EDs) are increasingly working together after‐hours, creating emergency care access points (ECAPs). This is one access point for out‐of‐hours emergency care for all patients.^3^, ^4^ At an ECAP, GPs at the GPCs are responsible for triage.^5^, ^6^ Triage will determine whether patients will be seen by the GP or a specialist at the ED. Before the implementation of ECAPs, patients could visit the ED on their own initiative. This gatekeeping function of GPs is seen as efficient patient care.^3^

The implementation of ECAPs has redirected many patients from the ED to the GPC, the majority being trauma cases with musculoskeletal complaints.^3^, ^5^, ^7^ In these cases, imaging is often indicated, with X‐rays being the most common diagnostic tool.^5^, ^8^, ^9^

At the moment, the possibility of GPs to request an X‐ray varies per ECAP. Less than 20% of the GPCs in the Netherlands are offered the possibility to request X‐rays for their patients without having to refer them to the ED.^10^ Expanding imaging possibilities to GPCs might be more efficient, since not every patient that receives an X‐ray needs to be treated in the ED. Besides, with increasing ED numbers of older patients with more comorbidity and an increase in ED crowding, reducing nonurgent patients at EDs is crucial.^1^, ^3^ Consequently, questions are rising regarding which imaging possibilities GPCs at ECAPs should have access to. Only one study has previously examined the effect of imaging facilities for GPCs and concluded that access to radiology could prevent unnecessary referrals to the ED.^11^ Additionally, the aim of our study was to explore patient flow and possible reductions in ED referrals at an ECAP with X‐ray imaging access for GPs where the facilities already exist for years. In this study, we, therefore, took advantage of a naturally occurring situation. The hypothesis was that the majority of the patients needing X‐rays were examined and treated at the GPC. Secondary objectives were type of X‐rays and injuries.

## 2 METHODS

### 2.1 Study design and setting

This retrospective cohort study looked at all patients that visited an ECAP and had an X‐ray imaging requested by the GP at the GPC between January 1, 2014 and December 31, 2014. There were no exclusion criteria. General practitioner cooperatives use the Netherlands Triage System for triaging patients.^12^ Triage can be performed by telephone or physically at the GPC. The majority of patients phone the GPC where they either get an advice by phone, an appointment at the GPC, or a home visit by the GPC or the ambulance services in urgent situations. A minority of patients physically self‐refer to the GPC, where they are triaged according to the Netherlands Triage System and allocated to the GPC or the ED. The researched GPC was part of an ECAP located at a general city hospital in the Southeast of the Netherlands, serving a population of around 325 000 people. Out‐of‐hours care was provided at the GPC between 5 pm and 8 am on weekdays and 24/7 on weekends and national holidays. During out‐of‐hours care, this GPC had access to the radiology facility of the hospital to request X‐rays between 5 pm and 10 pm on weekdays and between 8 am and 10 pm during weekends. The acting radiologist reviewed the X‐rays on a regular basis and informed the GPC about the results. After 10 pm, patients needing X‐rays were referred to the ED. In the Netherlands, this is similar to the daily practice of GPs during office hours, where GPs can also request X‐rays and are informed by the acting radiologist about the results. To look at the effect on patient flow, we also gathered the overall number of patients presenting to the GPC and ED in 2014. The nationally recognized medical ethical committee of the Catharina Hospital granted institutional review board exemption. There was a disclaimer for our researched patient population to make them aware that their data could be used for research. Patients could always object to the use of their data, which in our study, did not happen.

### 2.2 Data collection

The registration database of the radiology department uses a coding system for every requested X‐ray. This system was used to select all patients that received an X‐ray requested by the GPC during the study period. Hospital records were collected for additional information, including sex, age, number and type of X‐ray, X‐ray abnormalities, referral to the ED, and treatment.

### 2.3 Data analysis

All data were anonymized before analysis. We made an overview of the outcome of X‐rays and referrals to the ED. As some patients visited more than once, patients were analyzed per visit. X‐ray findings were stated negative when the X‐ray(s) revealed no abnormalities (interpreted by the radiologist) and stated positive when one (or more) of the X‐rays revealed abnormalities. For data analysis, we used IBM SPSS Statistics Version 23. Descriptive statistics (totals, medians, interquartile range) were used to describe patient characteristics and patient flows.

## 3 RESULTS

### 3.1 Patient characteristics

Between January 1, 2014 and December 31, 2014, a total of 2243 patients presenting to the ECAP and treated by the GP received 2663 X‐ray examinations (Table 1). The mean age was 31 years, with an equal distribution of sex (48% male). The majority of the patients received one X‐ray (83%) and the most frequent indication was suspicion of a fracture (91%). A total of 36 305 patients visited the GPC in 2014 and 31 902 patients presented to the ED in the same timeframe.

**Table 1:** General practitioner patient characteristics, 2014

Characteristics	Total Group
Total number of patient visits	36 305
Total number of patients receiving an X‐ray, N^a^	2243
Total number of X‐rays, N	2663
Number of X‐rays per patient per visit, N (%)	
1	1866 (83.1)
2	341 (15.2)
3	30 (1.3)
4	5 (0.2)
5	1 (0.1)
Sex, N (%)	
Men	1070 (47.7)
Women	1173 (52.3)
Median age, years (range)	24 (0‐97)
X‐ray positive, N (%)	
Yes	830 (37.0)
No	1413 (63.0)
Referral, N (%)	
Yes	726 (32.4)
No	1517 (67.6)

a Total number of patients that received an X‐ray at the GPC.

### 3.2 X‐ray findings and referrals

**Figure 1:**
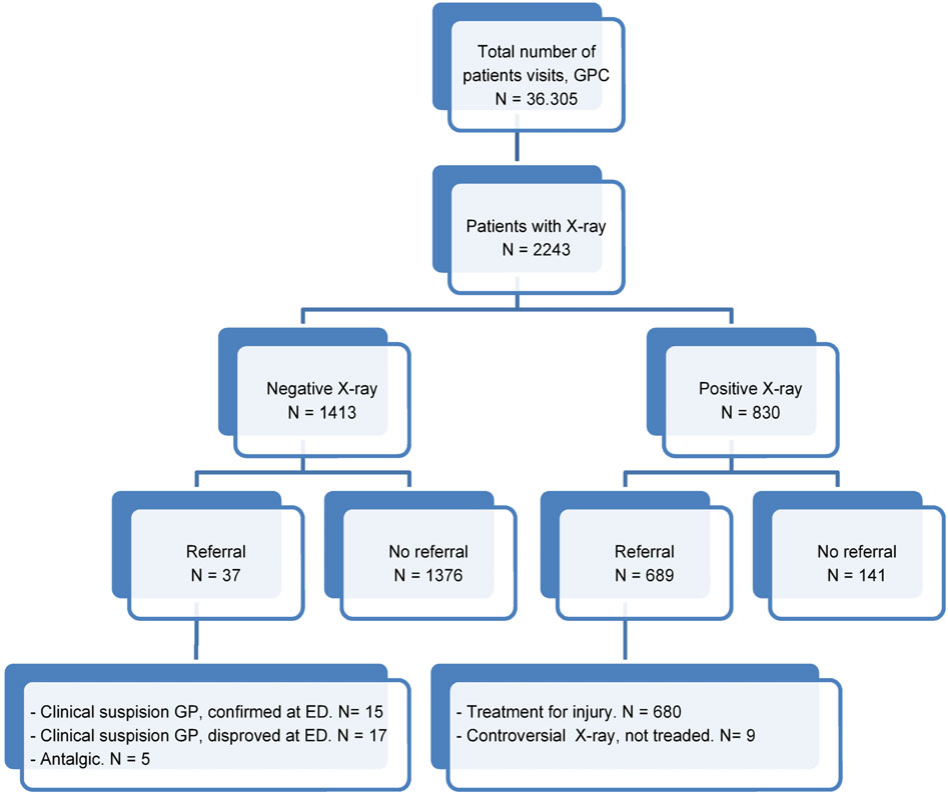
X‐ray outcome and patient flow to the ED, 2014. ED, emergency department; GP, general practitioner; GPC, general practitioner cooperative

A total of 1517 (68%) patients that received an X‐ray were treated at the GPC without an ED referral. Of these patients, 1376 (91%) had negative X‐ray findings and 141 (9%) had positive X‐ray findings (Figure 1). A total of 726 (32%) patients were referred to the ED, of which 689 (95%) patients had positive X‐ray findings and 37 (5%) patients had negative X‐ray findings but still a suspicious injury.

#### 3.2.1 Positive X‐ray findings

**Figure 2:**
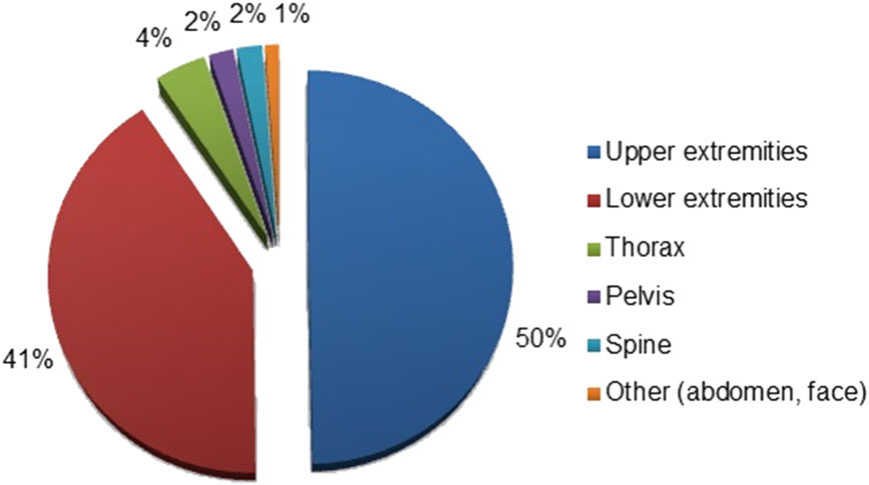
X‐ray examinations (N = 2663)

A total of 830 (37%) patients had positive findings on X‐ray (Figure 2), of which 689 (83%) patients were referred to the ED. The majority of the referred group, 680 patients (99%), was treated for a traumatic injury at the ED, which were mainly fractures of the arm (42%) and lower leg (37%). A minority (12%) was referred to the ED for other diagnoses, for instance, pneumonia. Nine patients (1%) had a controversial X‐ray and were stated negative at the ED after physical examination. The 141 patients (17%) with positive X‐ray findings that were treated solely at the GPC either had a controversial X‐ray outcome or were examined with small fractures of the distal extremities, for example, phalanx fractures, avulsion fractures of the ankle and foot, a fracture of the clavicle, or acromioclavicular joint injuries.

#### 3.2.2 Negative X‐ray findings

A total of 1413 (63%) patients had negative findings on X‐ray, of which 37 patients (5%) were still referred to the ED. This was either due to a suspicion of injury or for pain management. In 15 cases, the injury was confirmed at the ED, mainly being a suspicion of a scaphoid fracture. In 17 cases, no injury was found. Five patients were referred for a cast as part of pain management.

### 3.3 X‐ray examinations

The majority of requested X‐rays were for the upper (N = 1332, 50%) and lower extremities (N = 1092, 41%), namely, the wrist (N = 336, 13%), ankle (N = 415, 16%), and foot (N = 481, 18%), respectively (Figure 2).

## 4 DISCUSSION

The majority of patients (68%) that visited the GPC at the ECAP and received an X‐ray were examined and treated at the GPC without being referred to the ED. Of the patients referred to the ED, 95% had a definitive diagnosis by X‐ray and only 5% of patients needed a second opinion at the ED for a final diagnosis. The most frequent indication for an X‐ray was suspicion of a fracture, mainly of the distal extremities.

To the best of our knowledge, there is only one previous study that examined the effect of imaging facilities on patient flow at an ECAP where GPs have the possibility to request X‐rays without having to refer patients to the ED. The study by Rutten et al^11^ examined the outcomes and referrals to the ED in different access models for radiology and concluded that access to radiology by the GPC at an ECAP had benefits for patients and providers. Patient characteristics, injuries, and referral rate were comparable to our results. The GPC with limited time frames for radiology access had a referral rate of 38% compared to the 32% we found. Additionally, our study shows that of all patients that received an X‐ray at the GPC, 1517 patients were treated at the GPC and not referred to the ED. Studies show that GPCs make sensible use of the possibility of requesting X‐rays.^11^, ^13^ It is, therefore, plausible to assume that on an annual visit of 31 902 ED patients during the same timeframe, this has led to an estimated reduction of 4.5% of annual ED patients. This reduction was achieved with limited timeframes for GPs to request X‐ray imaging. Expanding X‐ray facilities at ECAPs throughout all opening hours is likely to reduce referrals even further.

With only 24% of the EDs working as an ECAP and less than 20% of these ECAPs having access to X‐ray diagnostics, the majority of patients that require X‐ray imaging are still being referred to the ED.^1^, ^10^, ^11^, ^14^ Not only will the change of patient flow reduce the numbers of ED patients but it will also reduce the overall length of stay of ED patients and possibly reduce health care costs.^15^ For self‐referring patients in the Netherlands, a consult at the ED is 3 times more expensive than a consult at the GPC.^16^ Furthermore, specialist care results in more diagnostic tests and more out‐patient follow‐ups than primary care, further reducing the costs.^17^ Collaboration of radiologists in out‐of‐hours care could further be cost‐saving by minimalizing additional out‐of‐hours shifts.

Without a control group in our study, we cannot exclude the risk that implementing X‐ray facilities at ECAPs might create an induced demand with general practitioners requesting more X‐rays. Nevertheless, multiple studies have shown the opposite. Implementations of ECAPs resulted in fewer X‐ray examinations, also when this was available for GPCs.^18^, ^19^, ^20^ Furthermore, there was no difference in risk assessment and indications for X‐ray requests between access and no access to radiology for GPCs.^11^

With increasing numbers of GPCs in Europe and multiple countries facing the problem of ED crowding and nonurgent self‐referring patients, our results could be of importance internationally.^1^, ^2^, ^10^, ^14^ There is one study in Norway that examined X‐ray facilities for GPCs and showed that only 13% of the GPCs had the possibly to request X‐rays in out‐of‐hours care, compared to 19% in the Netherlands.^10^, ^21^

This study had some limitations. Because of the retrospective study design, there is no follow‐up over time. Therefore, referrals to the outpatient clinic can be missed, although this is unlikely to result in a lower referral number to the ED. Furthermore, we only looked at patients who actually received an X‐ray. We did not look at patients who received no diagnostic imaging and where injuries could have been missed. Nevertheless, previous studies showed that the treatment of patients at the GPC maintained the safety and quality of care, and missed injuries were equal at the ED and GPC.^18^, ^19^ The observational design has the risk of not recognizing factors outside the ECAP that might have contributed to the results. Since this was a naturally occurring implementation, there was no before‐after study.

Implementation of X‐ray facilities for GPs at ECAPs is similar to the daily practice of GPs. Therefore, there will be no change for GPs in the medical procedure. There is an increase in patient load at ECAPs and this leads to a higher workload for GPs. Whether this will influence the quality of physical research and thereby, quality of X‐ray requests, is unknown. Another possible effect of X‐ray implementation can be an unnecessary treatment delay for patients who were first seen at the GPC. Further research should, therefore, focus on expanding X‐ray facilities for GPs and its influence on quality of X‐ray indications but also on the logistics of which patients can receive an X‐ray at the GPC and which ones should be directly referred to the ED. Economically, there will be a shift of health care costs towards the GP. The possible effect of this shift will variate internationally because of different health care systems.

In conclusion, the majority of patients (68%) that visited the GPC at the ECAP and received an X‐ray were examined and treated at the GPC. This resulted in a substantial decrease of ED patients, suggesting that X‐ray facilities for general practitioners at an ECAP will likely change patient flow and have a positive effect on ED crowding. Implementing 24/7 X‐ray facilities at all ECAPs will further enhance these effects.

## CONFLICT OF INTEREST

The authors have no support, funding, or competing interests to report.

## AUTHOR CONTRIBUTIONS

Conceptualization: Donna van den Bersselaar, Wendy Thijssen, Maaike Maas

Data Curation: Donna van den Bersselaar

Formal Analysis: Donna van den Bersselaar

Investigation: Donna van den Bersselaar

Project Administration: Donna van den Bersselaar, Wendy Thijssen

Resources: Wendy Thijssen, Maaike Maas

Supervision: Wendy Thijssen

Validation: Wendy Thijssen, Maaike Maas

Visualization: Donna van den Bersselaar, Wendy Thijssen, Maaike Maas

Writing—Original Draft Preparation: Donna van den Bersselaar

Writing—Review and Editing: Donna van den Bersselaar, Wendy Thijssen, Maaike Maas
